# Neoadjuvant chemotherapy in a rare type of very locally advanced sinonasal carcinomas – long-term results from a tertiary care centre

**DOI:** 10.3332/ecancer.2023.1549

**Published:** 2023-05-11

**Authors:** Saswata Saha, Vijay M Patil, Vanita Noronha, Nandini Menon, Ajay Kumar Singh, Mannavi Suman, Amit Agrawal, Satvik Khaddar, Shatabdi Chakraborty, Prathamesh S Pai, Devendra A Chaukar, Pankaj Chaturvedi, Sarbani Ghosh Laskar, Kumar Prabhash

**Affiliations:** 1 Department of Medical Oncology, Tata Memorial Hospital, Homi Bhabha National Institute (HBNI), Mumbai 400012, Maharashtra, India; 2 Department of Head and Neck Surgery, Tata Memorial Hospital, Homi Bhabha National Institute (HBNI), Mumbai 400012, Maharashtra, India; 3 Department of Radiation Oncology, Tata Memorial Hospital, Homi Bhabha National Institute (HBNI), Mumbai 400012, Maharashtra, India; † Both the authors contributed equally and to be considered as first author.

**Keywords:** neoadjuvant chemotherapy, sinonasal undeifferentiated carcinoma (SNUC), sinonasal adenocarcinoma (SNAC)

## Abstract

**Introduction:**

Sinonasal carcinomas are a rare type of head and neck malignancy with various histologies. The outcomes of patients with unresectable locally advanced sinonasal carcinomas are poor. Hence, we performed this analysis to study the long-term outcomes of sinonasal adenocarcinoma (SNAC) and sinonasal undifferentiated carcinomas (SNUC) where neoadjuvant chemotherapy (NACT) has been given followed by local therapy.

**Methods:**

16 patients with SNUC and adenocarcinoma who received NACT were found eligible for the study. Descriptive statistical analysis was performed for baseline characteristics, adverse events and treatment compliance. Kaplan Meir methods were used for the estimation of progression-free survival (PFS) and overall survival (OS).

**Results:**

Seven (43.75%) adenocarcinoma and nine (56.25%) SNUC patients were identified. The median age of the whole cohort was 48.5 years. The median number of cycles delivered was 3 (IQR 1–8). The incidence of grade 3–4 toxicity (CTCAE version 5.0) was 18.75%. The response was partial response or better in seven patients (43.75%). Post-NACT 11 patients (*n* = 15, 73%) were eligible for definitive therapy. The median PFS was 7.63 months (95% CI, 3.23 – NA months) and the median OS was 10.6 months (95% CI, 5.2–51.5 months). Median PFS and OS for those who underwent surgery post-NACT versus those who did not undergo surgery were 36.267 versus 3.7 months (*p* = 0.012) and 51.5 versus 10.633 months (*p* = 0.190), respectively.

**Conclusion:**

The study shows a favourable role of NACT in improving resectability, significant improvement in PFS and non-significant improvement in OS after surgery.

## Introduction

Carcinomas of the nasal cavity and paranasal sinus are rare malignancies. It accounts for about 1% of all malignancies and approximately 3% of cancers of the head and neck [1]. Sinonasal carcinoma has multiple histological types and features, along with various grades of differentiation with the same histology. Sinonasal squamous cell cancer and intestinal type adenocarcinoma (ITAC) have the highest incidence, followed by other histological types [2, 3]. Adenocarcinoma represents the second most common histologic subtype, accounting for approximately 13% of all cases of sinonasal malignancy [4]. Sinonasal undifferentiated carcinoma (SNUC) comprises 3%–5% of all sinonasal carcinomas [5].

SNUC is commonly diagnosed when it already involves multiple adjacent areas. Surgery is the mainstay of treatment for sinonasal carcinomas. Complete surgical resection in the absence of adverse pathological features may obviate the need for postoperative radiotherapy (RT) in T1 patients only [6]. However, the tumour’s location close to important structures like the orbit and the skull base makes surgery difficult. A multimodality therapeutic approach is mandatory, which is in general based on complete surgical resection with postoperative RT [5]. In patients with positive or close margins, high-grade lesions, or other unfavourable histology, and/or intracranial and/or intraorbital extension, postoperative systemic therapy/RT should be considered [6]. The prognosis of patients with very locally advanced unresectable sinonasal carcinoma is generally poor, with 5-year survival being approximately 30% despite advances in treatment [3]. The inclusion of systemic therapy may offer improvements in the locoregional control rates, increase the probability of resection and reduce the frequency of distant metastases. Hence, we performed this analysis to study the long-term outcomes of SNAC and SNUC where neoadjuvant chemotherapy (NACT) has been given followed by local therapy.

## Methods

### Patient selection and treatment

**Figure 1.**
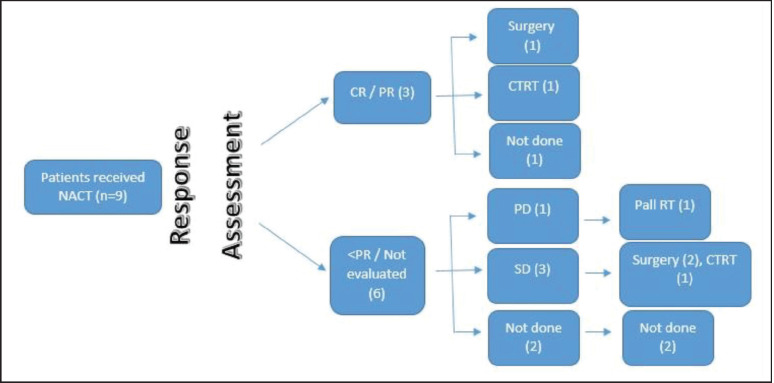
SNUC flow diagram.

**Figure 2.**
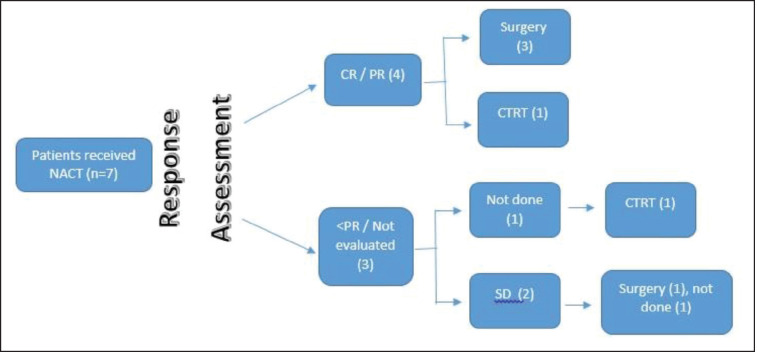
SNAC flow diagram.

The Medical Oncology Head and Neck Unit maintains a prospective database of patients undergoing NACT. This database was accessed to identify the patients with SNAC and SNUC who received NACT. These patients were seen in a multidisciplinary clinic and an algorithm of NACT followed by response assessment was decided. They were planned subsequently for 2–3 cycles of NACT. Post 2 cycles of NACT, patients were seen in a multidisciplinary clinic, and depending on response and performance status (PS), either curative intent therapy (surgery followed by adjuvant therapy/concurrent chemoradiation) or palliative intent therapy (palliative RT/palliative chemotherapy/best supportive care) was decided (Figures 1 and 2).

### Data collection

Patients’ demographic and baseline characteristic data were collected from electronic medical records. 16 patients of unresectable SNUC and adenocarcinoma received NACT and were found eligible from the database. Details regarding NACT and subsequent treatment were collected from the database. Adverse events during NACT were graded and reported as per CTCAE 5.0 (common terminology criteria for adverse events). Response assessments were done after scheduled NACT chemotherapy and responses were recorded using response evaluation criteria in solid tumours 1.1. Partial response was defined as ≥30% decrease in the sum of target lesions as compared to baseline while stable disease included <20% increase in the sum of target lesions where the partial response was not achieved. Patients with >20% increase in the sum of target lesions or the appearance of new target lesions were considered a progressive disease. The date of progression, death, or date of final hospital visit was recorded from the electronic medical record.

Statistical Package for the Social Sciences descriptive statistics has been performed. Descriptive statistical analysis was performed for baseline characteristics, adverse events and treatment compliance. The continuous variables were described in terms of the median with range while non-continuous variables were described in terms of percentages with 95% CI. Kaplan Meir methods were used for the estimation of progression-free survival (PFS) and overall survival (OS). PFS is defined as the time from diagnosis to progression, death due to any cause, or last date of the hospital visit. OS is defined as the time from diagnosis to death due to any cause or last date of the hospital visit.

## Results

### Baseline characteristics

16 patients with sinonasal cavity cancer were identified. The baseline details are shown in Table 1. The median age of the whole cohort was 48.5 years (IQR 41.75–56.75 years). The Eastern Co-operative Oncology Group (ECOG) PS was 0–1 in 15 (93.75%) patients and only 1 (6.25%) patient had ECOG PS 2. There were seven (43.75%) adenocarcinoma patients and nine (56.25%) SNUC or poorly differentiated patients. p53 status was evaluated by immunohistochemistry in histopathology specimens of only four patients (25%) and all were wild type. Out of these 16 patients, 10 patients (62.5%) were considered to have extensive disease, 2 patients (12.5%) had an oligometastatic disease and 4 patients (25%) were considered to have aggressive disease biology.

### Disease extent and reason for neo-adjuvant chemotherapy

The extent of locoregional spread is shown in Table 2. Regional lymph node involvement was seen in five patients (31.25%). An intracranial extension was seen in eight patients (50%), an extradural extension was seen in four patients (25%) and an intradural extension was seen in three patients (18.75%), respectively. Two patients (12.5%) had metastatic disease at baseline. The reason for NACT was extensive disease in ten patients (62.5%), oligometastatic disease in two patients (12.5%), and aggressive/high-grade tumours in four patients (25%).

### NACT details and compliance

Out of 16 patients at least 2 cycles of NACT were completed by 15 patients. The median number of cycles delivered was three (IQR 1–8). 13 patients (81.25%) received more than 2 cycles before locoregional treatment. NACT was not completed by three patients (one patient defaulted and in two patients stopped because of toxicity. The reason for NACT, chemotherapy regimens, and compliance are shown in Table 3.

### Adverse events

The incidence of grades 3 and 4 toxicity in accordance with CTCAE version 5.0 was 18.75%. There was no grade 5 toxicity seen. The details of adverse events are shown in Table 4.

### Response to neo-adjuvant chemotherapy

The response was evaluable in 13 patients (81.25%) after completion of NACT. The response was partial response or better was noted in seven patients (43.75%), stable disease in five patients (31.25%), progressive disease in one patient (6.25%), and not evaluated in three patients (18.75%). Details of the response to NACT are given in Table 5. The patient with baseline oligometastatic disease progressed in the right iliac bone and is the only patient with disease progression after NACT.

### Treatment received post-NACT

Post-NACT 11 patients (*n* = 15, 73%) were eligible for definitive therapy in the whole cohort of 16 patients. Treatment received was surgery in seven patients (43.75%), definitive chemo-radiotherapy in four patients (25%), and palliative RT in one patient (6.25%). One patient had progressive disease after NACT and was not suitable for any local treatment (received palliative RT). Details of treatment received post-NACT are depicted in Table 6.

The post-surgery residual disease was noted in all seven patients (43.75%). No nodal disease was noted in the post-surgery specimen. The margin status in the post-surgery specimen is given in Table 7.

### Survival analysis

**Figure 3.**
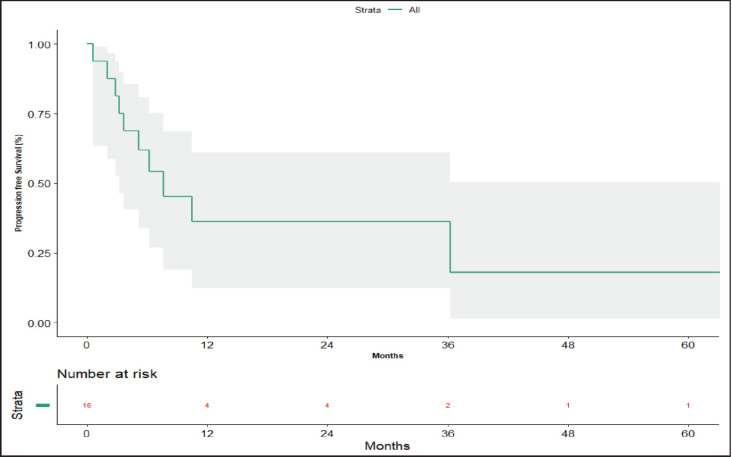
Progression-free survival.

The median follow-up period is 56.20 months (95% confidence limit 5.57 months – NA). At the time of data analysis, seven patients (43.75%) had progressed, two patients (12.5%) died without progression and four patients (25%) were lost to follow-up. The median PFS calculated was 7.63 months (95% CI, 3.23 – NA months) (Figure 3). The median PFS for those who underwent surgery post-NACT was 36.267 versus 3.7 months for those who did not undergo surgery post-NACT, which is statistically significant (*p* = 0.012).

**Figure 4.**
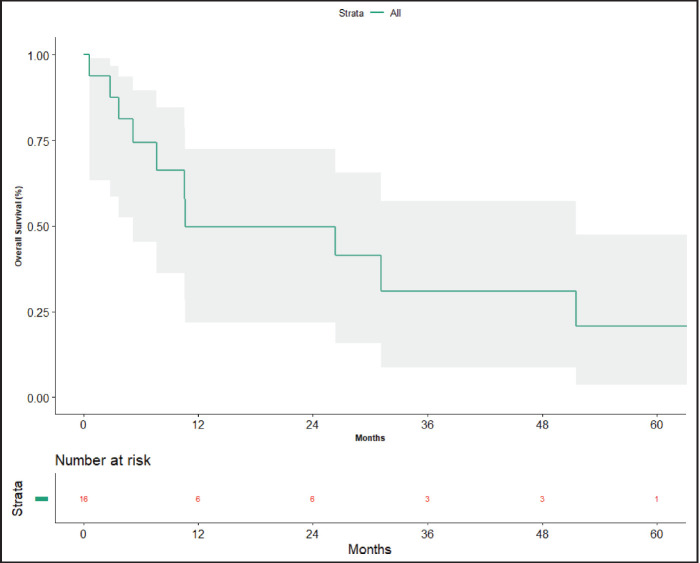
Overall survival.

**Figure 5.**
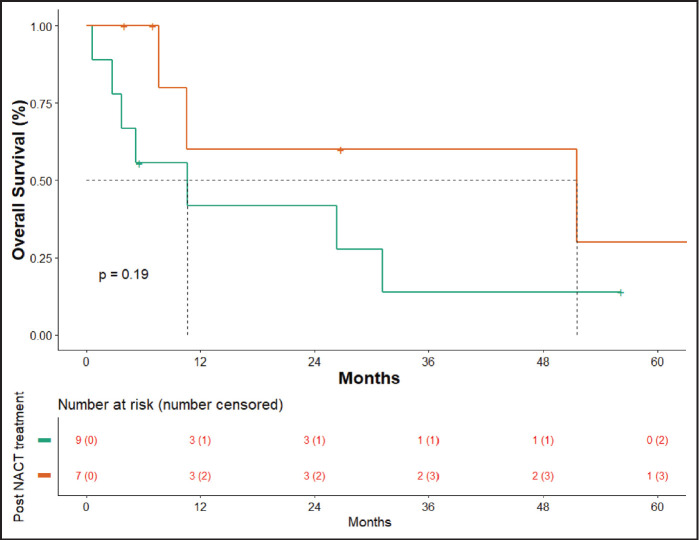
OS surgery versus no surgery.

At the time of data entry, four patients (25%) died and four patients (25%) were lost to follow-up. The estimated median OS was 10.6 months (95% CI, 5.2–51.5 months) (Figure 4). OS in patients who underwent surgery was 51.5 months (7.63 months – NA) versus 10.633 months (0.60–31.2 months) in those who did not undergo surgery post-NACT (Figure 5), which is numerically significant but did not reach a statistically significant value (*p* = 0.190).

**Figure 6.**
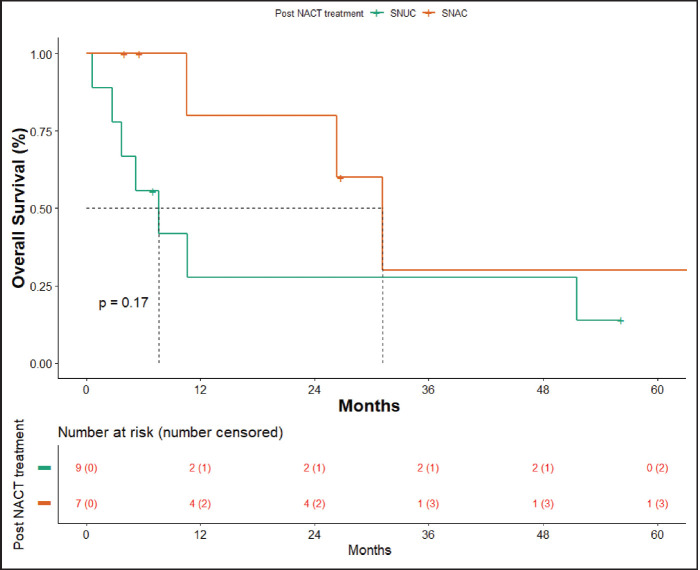
OS SNAC versus SNUC.

OS in patients with SNAC was 31.17 months (10.6 months – NA) and in patients with SNUC histology was 7.63 months (0.6–51.5 months), though not statistically significant (*p* = 0.17) (Figure 6).

## Discussion

Sinonasal carcinomas have various histologies. Squamous cell carcinoma is the most common histology. Other histologies of specific significance are esthesioneuroblastoma, sinonasal carcinoma with neuroendocrine differentiation, undifferentiated carcinoma, adenocarcinoma (intestinal and non-intestinal type), and other rare types. It is well known that the prognosis of locally advanced sinonasal malignancies varies according to histology and stage [7]. In this analysis, we discussed the role of NACT in unresectable SNUC and SNAC.

The factors affecting prognosis in SNAC as reported in various studies are tumour stage, intracranial involvement, lymph node involvement at diagnosis, and treatment modality of the primary tumour site [8, 9]. All patients in the study had stage Kadish B–D. Nodal involvement was noted in five patients (31.75%) at baseline and intracranial extension was noted in eight patients (50%). In three patients (18.75%) with Kadish B stage, NACT was decided before surgery because of the aggressive biology of the disease as poorly differentiated tumours have worse survival when compared to well or moderately differentiated tumours in various studies [10, 11], and in rest 13 patients (81.25%), NACT was given because of extensive disease.

Surgery with a wide resection margin is the mainstay of treatment for small primary tumours with low-grade histology, while surgery followed by radiation has remained a mainstay in the treatment of advanced disease [12–14]. Induction chemotherapy has been used in a few isolated studies reported till now.

Bossi *et al* [15] described the role of the beneficial outcome of multimodality treatment including systemic therapy for locally advanced sinonasal cancer. A study by Bjork-Eriksson *et al* [16] evaluated the role of induction cisplatin and 5-fluorouracil chemotherapy in 12 patients of non-adenocarcinoma sinonasal tumours. 11 patients achieved local control and after a median follow-up of 27 months, 10 patients were disease-free and alive.

In a study of 30 patients with ITAC , the cisplatin-5FU-leucovorin induction regimen achieved a pathological complete response (pCR) rate of 40%. The study also found that the presence of functional p53 protein was significantly related to the probability of obtaining a pCR [17]. In our study, p53 status was evaluated in four patients (adenocarcinoma histology) and all had p53 wild-type expression. Hence, no correlation was possible in this study. One patient (6.25%) underwent concurrent chemoradiotherapy post-induction chemotherapy and the rest three (18.75%) underwent surgery and had a residual disease in the post-NACT specimen.

A meta-analysis of published trials on SNUCs (*n* = 167 patients) showed that the addition of systemic therapy to surgery improved survival in patients with loco-regionally advanced disease [18]. A study by the University of Virginia group used cyclophosphamide, doxorubicin, and vincristine as an induction regimen in this subset of patients. In this study, induction chemotherapy and radiation followed by craniofacial surgery showed a 2-year OS of 64%, whereas in unresectable patients, the 2-year OS was only 25% [19]. Our study also showed a similar outcome in patients (both SNUCs and adenocarcinoma) who underwent surgery versus those who did not, the median OS was 51.5 versus 10.633 months, respectively. Being a retrospective review, the number of NACT regimens used was different and was chosen according to the feasibility of administration and PS of the patient.

In SNUC patients, Rosenthal and Barker [20] in their study of 16 patients reported an excellent 5-year OS rate of 63%. In this series, 8 out of 16 patients received induction chemotherapy before definitive local therapy. The study by Nunez *et al* [10] in SNAC showed a variable survival rate range from as low as 30% after 3 years to as high as 75% after 5 years [10]. In our study, the median PFS was 7.633 months and the median OS was 10.633 months only.

The limitations of our study are that it is a retrospective study from a single institution only. The chemotherapy regimens used as NACT were varied and hence the response rate. A significant number of patients in the study were lost to follow-up and no further details regarding them were available.

## Conclusion

The study shows a favourable role of NACT in improving resectability, significant improvement in PFS and non-significant improvement in OS after surgery following NACT. Thus, NACT should be included to improve resectability and hence survival in patients with unresectable locally advanced tumours and rare histological types of sinonasal tumours.

## Conflicts of interest

There are no conflicts of interest.

## Funding declaration

Nil.

## Figures and Tables

**Table 1.** Baseline characteristics.

Characteristics	Value
Age	48.5 years (41.75–56.75 years)
Sex Male Female	10 (62.5%) 6 (37.5%)
Co-morbidities Diabetes mellitus Hypertension	1 (6.25%) 1 (6.25%)
Addiction Chewing tobacco Masheri Smoking Tobacco and alcohol	2 (12.5%) 1 (6.25%) 2 (12.5%) 1 (6.25%)
ECOG performance status 0 1 2	5 (31.25%) 10 (62.5%) 1 (6.25%)
Baseline histopathology ITAC Nnon-ITAC Undifferentiated carcinoma/poorly differentiated carcinoma	5 (31.25%) 2 (12.5%) 9 (56.25%)
Kadish stage B C D	3 (18.75%) 7 (43.75%) 6 (37.5%)

**Table 2.** Extent of the disease.

Characteristics	Value
Nodal disease Yes No	5 (31.25%) 11 (68.75%)
Cranial extension Cribriform plate Intracranial Extradural Intradural Intraparenchymal	10 (62.5%) 8 (50%) 4 (25%) 3 (18.75%) 1 (16.25%)
Metastatic disease Bones Bone marrow No	1 (6.25%) 1 (6.25%) 14 (87.5%)

**Table 3.** NACT details.

NACT details	Value
Reason for NACT Aggressive histology/high-grade tumour Extensive disease Oligometastatic disease	4 (25%) 10 (62.5%) 2 (12.5%)
NACT regimen CAPOX/FOLFOX Carboplatin-etoposide/Cisplatin-etoposide TPF/Docetaxel-cisplatin Paclitaxel-carboplatin	4 (25%) 3 (18.75%) 7 (43.75%) 2 (12.5%)
Number of NACT cycles received 1 2 3 or more	1 (6.25%) 2 (12.5%) 13 (81.25%)
Compliance to NACT Completed Incomplete -Defaulted -Toxicity	13 (81.25%) 3 (18.75%) 1 (6.25%) 2 (12.5%)

**Table 4.** Adverse events as per CTCAE 5.0.

Adverse events	Any grade	Grade 3 or above
Nausea/vomiting	8 (50%)	–
Fatigue	7 (43.75%)	1 (6.25%)
Diarrhea	7 (43.75%)	1 (6.25%)
Oral mucositis	2 (12.5%)	–
Neuropathy	3 (18.75%)	–
Febrile neutropenia	1 (6.25%)	1 (6.25%)
Anemia	7 (43.75%)	–
Neutropenia	4 (25%)	3 (18.75%)
Thrombocytopenia	2 (12.5%)	–
Transaminitis	2 (12.5%)	–
Creatinine rise	–	–
Hyponatremia	10 (62.5%)	2 (12.5%)
Hypokalemia	3 (18.75%)	–

**Table 5.** Response post-NACT.

Response to NACT	Value
Partial response or better	7 (43.75%)
Stable disease	5 (31.25%)
Progressive disease	1 (6.25%)
Not evaluated	3 (18.75%)

**Table 6.** Treatment post-NACT.

Treatment post-NACT	Value
Surgery	7 (43.75%)
Concurrent chemo-radiotherapy	4 (25%)
Palliative RT	1 (6.25%)
Supportive care/defaulted	4 (25%)

**Table 7.** Margin status post-NACT.

Margin status post-surgery	Value
Close margin	2 (12.5%)
Positive margin	1 (6.25%)
Negative margin	4 (25%)
